# Diseased Meat and Its Consequences upon Our Health and Happiness

**Published:** 1880-10

**Authors:** Noah Cressy

**Affiliations:** Hartford Conn.


### ART. XVIII.—DISEASED MEAT AND ITS CONSEQUENCES UPON OUR HEALTH AND HAPPINESS.

AN ADDRESS DELIVERED BEFORE THE CONNECTICUT BOARD OF AGRICULTURE.

BY NOAH CRESSY, M.D., V.S. PH.D.
HARTFORD CONN.

The relations of man to the lower order of animals, zoologically considered, which of late has caused so much speculation among philosophers and naturalists, is equally interesting in a physiological and pathological point of view. The skeleton framework and internal organization of the higher mammalia are morphologically identical with that of man, and subserve the same purpose in the animal economy. The blood is similar in chemical composition, contains the same anatomical elements, and is subject to analogous changes in disease. Hence the liability of transmitting to the human system some virulent blood-poison or other morbid products through the medium of the animal food which we consume.

Consequently I believe that there is no subject of more importance to the public health or better calculated to enhance the cause of sanitary science than the practical study of diseased meat. And though many inquiries have been made in this direction and some valuable conclusions reached, yet in this broad field for scientific research the accomplished laborers are few. But the hour has come when the sanitarian and physician, in response to a public demand, must join hands with the veterinary profession to explore certain realms in the causation of disease and also more accurately survey those boundary lines in pathology which have served as a barrier to the progress of knowledge on this subject, and even now seem to separate the human maladies from those of our food-producing animals.

In fact the very condition of some of our meat supplies already indicates the solution of certain vexed questions on the origin of disease that have been a stumbling block to the medical practitioner. The investigation of this subject, therefore, in all its varied relations to our health and happiness, is a work of vast importance, and one which the age now demands, in behalf of sanitary science. Hence it will require for the achievement of these results not only the united efforts of professional and scientific men but the influence of the public purse and the sanction of our State authorities. Then may we hope to see a thorough system of veterinary inspection established in this country, that shall have full control of the public markets, and examine all suspicious animals before they are allowed to be slaughtered.

### NATURE OF DISEASED MEAT.

Few questions in dietetics have been more thoroughly discussed of late years, both by the rich and the poor, than the sanitary condition of animal food; and the differnnce of opinion on flavor and quality of meat is in a great measure due to habit and education. Our appetites are capricious, and as there is no accounting for taste; what suits one individual may fail to relish with another. But an unpalatable article of meat is not necessarily injurious. In fact, the flesh of animals suffering from a great variety of maladies may be eaten with impunity. Yet the flesh of some, without an apparent symptom of disease during life, may prove fatal when consumed by man. There are certain malignant maladies, also quite prevalent among some of our domesticated animals, that do not produce an unpleasant flavor in the meat for the palate of the epicure, but which, in the advanced stages, often render the carcass exceedingly hazardous for the butcher to dress.

All meat, therefore, from whatever source or condition of an animal it may come, that would cause sickness, disease or death in man if partaken as food, must be regarded in the light of sanitary science as diseased, and consequently unfit for human use in any form. Accordingly an article of meat possessing such qualities must come from an animal afflicted with some form of an infectious malady, the germs of which are contained in the flesh, and are liable to be transmitted.

Hence a disease in which a contagious virus is developed during its course, or a virulent principle generated in the blood, renders the meat from all animals thus affected exceedingly dangerous as an article of food. But meat is not materially affected by the entozic maladies of animals, unless the parasite in some stage of its existence makes its abode in the flesh and has not been destroyed by cooking.

In accordance with this definition there are but few diseases that absolutely render these animal supplies perilous to human happiness. Prominent among these may be mentioned malignant anthrax, hydrophobia, tuberculosis, small-pox, and two parasitic affections caused by the trichina spiralis, and the measle tape worms. But the other maladies, from which our slaughtered animals are liable to have suffered, may greatly impoverish the nutritive quality of the meat, and thus render it unpleasant in taste and general appearance, yet if the flesh contains no animal poison or other morbid products no harm can possibly come from its use when served upon our table. And even a diseased article, when thorougly cooked, may not prove injurious to one whose digestive powers are active.

It is not an easy matter, therefore, in all cases to decide whether meat is possessed of injurious qualities or not without a careful inquiry into the history of the article, or a microscopic inspection. Trichinous pork is an example of this kind; and of the many fatal cases on record, none of the victims ever suspected the meat until a peculiar form of sickness made its appearance, involving a number of individuals who were known to have partaken of the same. This is also true of black-leg veal many times, and of other fine looking specimens of meat that are affected with anthrax poison.

Many varieties of diseased meat, however, are so palpable that even by the dexterity of the butcher's art it is impossible to disguise them. The tuberculous deposits upon the pleural membrane lining the chest cavity, causing the lungs to adhere to the ribs, or along the internal walls of the abdomen, are sufficient evidence to condemn the carcass. Measly pork and beef are also easily detected by the unaided eye; but the parasitic contamination of such meat is often overlooked, and consequently there is a fine opportunity for a tape worm to become initiated in all who may partake of it.

The extent to which the different kinds of diseased meat are liable to be used will depend in a great measure upon the comparative frequency with which these infectious maladies occur among the live stock in a given locality; and the more insidious the nature of the disease the greater is the liability of animals being slaughtered that are more or less affected. Hence a brief description of some of the more common forms of suspected meat, with a review of the pathological conditions of the disease in question, will best serve our present purpose, and possibly throw a gleam of sanitary light on this much neglected subject.

### CONSUMPTIVE BEEF.

The meat from cattle affected with tuberculosis is not unfrequently seen in American markets, especially in our larger cities, and even in country towns. Yet, owing to the lack of public appreciation of any sanitary police measure to control such traffic, little or no complaint is made when we are served with consumptive beef. Five years ago, after repeated opportunities for observation on this subject, I called your attention to the prevalence of this malignant malady among our dairy stock, that I believed was publicly almost unknown. And I now affirm with renewed assurance in a pathological point of view, that its baneful consequences to our health and happiness from the use of meat and milk are not surpassed in the whole catalogue of contagious affections.

As this disease is comparatively new to the veterinary profession, its clinical history and pathology has not received that attention which the subject now demands. In fact, few are aware to-day of the extent to which this insidious malady prevails, but the rapid strides which it has made and the hold it has already gained on our stock, observes a veterinary author, renders it one of the most important questions affecting the well-being of the bovine species.*

The contagious nature of tuberculosis, as shown by recent experiments on animals, can no longer be doubted, and it is now conceded by comparative pathologists that the bovine form of this disease is identical with that of man. Consequently there is great liability of its transmission, either by inoculation or ingestion. In fact, it has repeatedly been produced in rabbits, Guinea-pigs, and calves by feeding them with tnberculous matter. And Prof. Gerlack of the Berlin Veterinary School claims,† as the result of his researches, that this disease in cattle is very infectious, that the presence of a specific virus is evident, and that even the flesh of such diseased animals under certain circumstances, and also the milk, possesses infective properties, though to a less degree than the cheesy matter from the lungs.

There is evidently a strong predisposition in neat stock to this disease, and they are more frequently affected than other domestic animals. The temperament and physical confirmation undoubtedly contribute much to its development; for animals of a phlegmatic type, with an attenuated form, long limbs, and narrow chests are usually the first victims of the malady. Breeders

---

* The Four Bovine Scourges, with an Appendix on the Inspection of Meat, etc., by Thomas Walley, M. R. C. V. S., Edinburgh, 1879.

† The Veterinarian, London, March No., 1875.

should therefore strive to avoid the possibility of transmitting such diseased qualities. It is more frequent in cows than in oxen, and especially those kept in dairies for a length of time. Hence lactation is believed to be a predisposing cause. The condition also in which animals are kept are no small factors. The cold, damp sheds, the dark, underground stables, and other ill-ventilated abodes, as well as the character of the food, all conspire to rekindle those constitutional taints into morbid activity.

That tuberculosis is rapidly on the increase no well-informed veterinarian can deny. It ranks among the few great scourges of the land; and though our losses, thus far, in live stock property have been largely due to other plagues which sweep their victims off in a manner to be seen by all, yet the ravages of this disease can only be realized, as Prof. Walley of Dick's Veterinary College* observes, when we take into account the vast deterioration, the slow but certain decimation of many of our best herds, the destruction of our animal supplies, and also the danger to human life which can no longer be considered chimerical. Still there are many who from want of knowledge on the subject may even despise the pathological significance of this fell destroyer and thus ignore its deadly meaning, but when we see thousands of these tubercular deposits in a single slaughtered animal, we are forced to conclude that the use of such meat can in no way promote our happiness. Thus we have in every form of tubercle an implacable and destructive foe, and, in fact, there is no other morbid product known that is so *protean* in the number of functional derangements to which it may give rise in the animal economy.

If we inquire further into the causes of the increased susceptibility to the infection, as seen more especially in our thoroughbred stock, we shall find that heredity and multiplied consanguinity play no menial part. Any physical weakness which the sire or dam may possess is liable to be transmitted to the immediate progeny, but if one generation escapes, the trouble may appear in the next, in accordance with the well–established principle of atavism. Diseased conditions are also inherited; and I believe that there is no predisposing cause which exercises such a potent influence in the production of tuberculosis as the pernicious system of in-and-in-breeding. Thus from parent to offspring, from one generation to another, we often see the fatal tendency transmitted in unbroken succession, and the more complicated the relationship becomes, the greater is the virulence of the resulting products. In spite, therefore, of the many palpable examples of this broken law, some breeders still pursue, year by year, the suicidal policy of clinging to *one strain*, regardless of the impending consequence. And Prof. James Law, of Cornell University, when speaking of consanguinous unions, says, " That the esteemed qualities have been preserved, strengthened, and increased in this way there can be no doubt, but there can be just as little doubt that any inherited weakness or disease has been often transmitted and even intensified. I could mention particular families in our highest priced breeds in which tuberculosis has become a fixed character." And further on he observes that " excessive weakness and stupidity of the young is another common result of in-breeding."*

In 1865, Prof. Villemin of the Val-de-Grace Hospital, Paris, having conceived that human consumption in certain cases might be due to a specific virus introduced into the system, resorted to a series of experiments on animals to test the question. He was the first to demonstrate the contagiousness of tuberculosis by inoculation. Rabbits and Guinea-pigs were selected, and the material employed was from the human lung. Inoculations were made in various parts of the body, but the results were uniform and of a serious character. Many of the creatures died, others, lingering in a depressed state, were killed when well marked tubercular deposits were found in all, especially in the lungs, and with more or less infiltrations in the other organs, thus showing that the disease had been transmitted.

These results which gave him so much renown as a pathologist led him to experiment with tubercular matter from other animals. Desirous, therefore, of testing the nature of the disease in cattle, he inoculated a rabbit with matter from a cow. The

* Report of the Am. Public Health Association, New York, 1875, vol. 2, p. 250.

animal became emaciated, and in six weeks was destroyed. Its lungs were filled with hard, tubercular masses, and some of them had taken on a cheesy aspect in the centre. The other organs of the body were affected in a similar manner as those in the previous experiments. Hence he concludes that bovine phthisis is identical with that of man.

Dr. Villemin has likewise demonstrated that the tuberculous matter produced artificially by inoculation possesses the same power of tranmissibility as when the malady arises spontaneously—thus proving conclusively that in tubercle resides a special elaborated virus which does not lose its identity by several removes, the same as small-pox.* This view of the subject is corroborated by the pathological researches of Dr. Lionel Beale, of London, the celebrated microscopist, who declares that tubercle is a minute particle of living matter, and if inoculated under favorable circumstances it is almost sure to grow, multiply, and produce other morbid cells like that from which it was derived.† And furthermore, Villemin has always considered tuberculosis a *specific* malady, for he found that a very small wound and an inconsiderable quantity of matter used was a manifest proof that the intensity of disease is independent of the *quantity* of the matter inoculated, and that the number and extent of the internal lesions have no relations to those at the seat of the puncture.

A disease, therefore, that can be transmitted from one animal to another by inoculation and thus an identical virus reproduced is, strictly speaking, *contagious*. But further and more convincing proof on this subject has been furnished by Prof. Chauveau, of the Lyons Veterinary School, who for years has been experimentally studying the intimate pathology of the various contagia. The success of these researches has afforded some startling results pertaining to the use of diseased meat. The discovery also that certain rich virulent matter can infect as readily through the digestive organs as by any other channel, has given him a world-wide reputation. And his well-designed experiments on

---

* See *Veterinarian*, for January, 1875.

† The Microscope in Medicine, fourth ed., London, 1878, pp. 329–38.

cattle, which he instituted in 1868, have settled forever among comparative pathologists the question of the virulency of tuberculosis.

He purchased four calves the 18th of September, from a locality where this disease was unknown, which upon rigid examination were found to be in fine, healthy condition. The next day he administered an ounce of tubercular matter from an old cow's lung, including the hard and soft varieties, prepared in the form of a drench and given in divided doses. The first one, a year old, began to lose condition in about a fortnight, the respirations were quickened, though the appetite remained unimpaired. On the 5th of October he gave this calf another dose, but of different and more recent matter, and within a week the symptoms of tuberculosis were apparent. Emaciation proceeded rapidly, the coat became rough and staring, and the animal had occasional fits of coughing, especially after drinking.

The second calf, six months old, had on the fourth day a profuse and fœtid diarrhœa, but of short duration, and the animal remained apparently healthy for three weeks. But the characteristic symptoms as in the other case soon appeared, with enlargement of the glands about the throat. The third one of the same age, having shown no signs of disease, was drenched again October 9th with another kind of matter, but this calf longest resisted the action of the virus, and not until the 25th was there any appreciable derangement of health ; but from that time, however, the phenomena of tubercular infection ensued with amazing rapidity, and in a week the calf could scarcely be recognized.

At the close of the experiments, November 10th, the miserable aspect of the three infected creatures when contrasted with the thriving condition of the fourth, left no doubt in the mind of even the casual observer as to the changes that had taken place. The post mortem examinations revealed a perfect generalized form of tuberculosis, with the local lesion of the bowels, *tabes mesenterica*, shown in a marked degree, some of the glands being as large as a man's fist. The morbid deposits in the chest cavity also were none the less remarkable. The lungs were studded with crude tubercles, some forty in number, varying in size from a pea to a filbert. The bronchial glands were also involved, but the liver, spleen, and kidneys were not affected.

Thus in the space of fifty-two days we have three typical examples, nearly uniform in appearance, of the artificial production of this malignant malady through the digestive organs. In presence of these facts, therefore, I trust that all inquirers after the truth of this matter will be forced to conclude with our illustrious pathologist that the virulence and contagious properties of tuberculosis are now demonstrated beyond a doubt. And the fact that bovine animals have contracted this disease through the agency of the feed gives us an additional source of danger, for creatures confined in the same stable or pasture, and drinking from the same ponds or troughs, are constantly liable to swallow some of this virus in the mucus discharges from the nostrils of their affected comrades. In fact, it is never safe to put another animal in the same stall where one has sickened and died of this complaint without thoroughly renovating the apartment. Nor would I allow an affected creature to mingle with the healthy stock about the yard.

The observation of Dr. Grad,* veterinary surgeon at Wasselonne, Alsace, on the spread of this disease by contaminated stalls, are very conclusive. On different occasions owners had informed him that they had lost several animals from consumption *in the same stall.* At first he did not attach much importance to the matter, but one day, when visiting the stables of an extensive farmer in Leinheim, he was informed that annually for the last five years, one of the cattle had died of tuberculosis in a certain stall. The last one he had the opportunity of examining, which had been there but ten months, but had all the symptoms of the malady, greatly emaciated, and troubled with a cough. Dr. Grad's attention was strongly aroused at such a state of things, and to test the matter scientifically he was allowed to select an animal for an experiment. Accordingly he chose from another stable a three-year-old heifer, in calf, that was to all appear-

* See Fleming's able memoir on the history of these investigations in the 48th and 49th Vols. of *The Veterinarian.*

ances perfectly healthy. She was bred on the farm, had never been unwell, never coughed, and none of her progenitors had ever been affected with phthisis. The cow remained quite well until after calving, when a slight cough appeared; but it increased in frequency, emaciation gradually set in, with all of the symptoms of tuberculosis, and in twelve months the creature was a mere shadow of her former self. The evidence, therefore, in support of this mode of infection Grad could no longer resist, as this was the sixth case that had occurred in this stall. Hence he very naturally inferred that the disease was probably transmitted by the ingestion of tuberculosis matter expectorated by the cattle which had previously occupied the place.

The extension of the malady by cohabitation is therefore always liable to occur when animals are so arranged in the stable that the sick and healthy ones can get their heads together, or feed from the same manger. The hay may thus become contaminated, and the infection takes place through the digestive organs. The expired air also is not unfrequently so laden with virulent matter, especially in the advanced stages, that it is not safe for another animal to inhale it. This mode of transmission, which was first suggested by Dr. Morgagni,* more than a hundred years ago, and has found many advocates among physicians and veterinarians, has now been confirmed by the experiments of Dr. Tappeiner, of Meran, in causing animals to inhale the fine particles of tubercular matter from the air of a room in which the virus had been evaporated by a steam atomizer. Out of eleven puppies experimented on, ten showed well-marked miliary tubercle in both lungs on being killed within twenty-five to forty days—thus proving that this disease is contagious by the breath.

The relation of bovine tuberculosis to public hygiene was probably first suggested by Prof. Chauveau, who twelve years ago had already indicated the real source of danger from the use of consumptive meat and milk. But no one has done more to promulgate these investigations, or has contributed more to the

* Nature and Cause of Diseases. Lond., 1769.

advancement of veterinary education in this direction, than George Fleming, F. R. C. V. S., inspecting veterinary surgeon to the British army, and the learned editor of the London *Veterinary Journal*, who, by his encyclopedic writings, has now become an acknowledged authority on the subject.† And in a recent editorial,‡ he says, "That the tuberculosis of cattle is a transmissible disease, and can be conveyed not only to animals of the same, but also to those of other species in various ways, is now an established fact, upon the recognition of which we have for many years insisted; and since we first called attention to it, some of the best pathologists in Europe have furnished additional testimony as to the readiness with which this transmission takes place, not only by inoculation or ingestion, but also, it would appear, by cohabitation of diseased with healthy animals.

Early in 1879, Prof. Colin, of the Alfort Veterinary School, contributed a series of observations with regard to the communicability of the disorder; several German and Italian authorities have also published their clinical experience in this direction; and lastly, we have the celebrated Prof. Orth, of Gottingen, furnishing the results of his researches and experiments. All these are only confirmatory of what we have already stated, but this confirmation is not without its value. Dr. Orth's experiments once more demonstrates that the transmission of bovine tuberculosis is possible between different species of animals, and he also points out the complete analogy or rather perhaps identity, which exists between it and the disease in man.

In his experiments, fifteen animals were fed with tuberculous matter from a diseased cow, and nine of those were infected, of which four died. The remaining five becoming extremely emaciated, were killed. On examination nearly all the organs of the body were found involved in tuberculosis. In all the lungs were affected, but the serous and mucus membranes, the lympathic

---

† See his Manual of Veterinary Sanitary Science and Police, in two vols., 8vo, illustrated. Lond., 1875.

‡ *Veterinary Journal*, Dec., 1879.

glands, the liver, spleen, kidneys, and omentum, were infected in different degrees. And consequently the transmissibility of this affection to animals being proved, he insists that its transmission to man is possible.

The meat of animals affected with this disease should not be used, for any organ or texture in which tubercle has been deposited is surely a dangerous article of food. Much will depend, however, upon the severity of the case and extent of the morbid changes that have taken place. Thus, from what is known in relation to the pathology of this virulent malady, we should at once interdict the sale of consumptive beef and milk, especially in the advanced stages of the disease, when the glandular tissues have become involved. As the commencement of phthisis is often so insiduous in the human subject, and so very difficult at times to arrive at the exciting causes, it is to be feared, at least, that one of the sources of its invasion, according to recent experiments, is to be referred to the utilization of the carcass, but more particularly the milk of such diseased cattle, as food.

There is every reason therefore, says Fleming, to prohibit the use of milk from cows affected with tuberculosis, and especially for infants, who mainly rely upon this fluid for their sustenance, and whose powers of absorption are very active. Even if it did not possess infective properties, its deficiency in nitrogenous elements, fat and sugar, and the increased proportion of earthy salts, would alone render it an objectionable article of diet. In fact, it has long been known that it was liable to produce diarrhea and debility in infants; but though many children fed on such milk have died from tuberculous or a localized type of it in the bowels known as *Tabes mesenterica*, the part probably played by this liquid in its production has rarely been suspected.*

The recent investigations of Prof. Otto Bollinger of the University of Munich, on the artificial production of tuberculosis as induced by the consumption of diseased milk, has thrown additional light on the subject. He claims that the milk of such

---

* Paraphrased from his Sanitary Police, Vol. 2, page 396.

animals has a pre-eminently contagious influence, and reproduces the disease in other animals experimented on from that point of view. He believes also that such milk, even when boiled, still retains its injurious properties. Further, he maintains that beyond doubt the tuberculosis of the human subject, though not completely identical with that of the cow, is yet strictly analogous to it, and that consequently the wide prevalence of tuberculosis in the native herds, at least five per cent. of which are affected, is a standing danger to health of the community. Seeing the enormous mortality from consumption, more especially in towns, Prof. Bollinger believes it to be of the utmost importance to urge upon all classes, and particularly upon farmers, the absolute necessity of taking every possible means of stamping out the disease among cattle. Meanwhile some measure of safety may be secured by the rigid exclusion of all diseased stock from town dairies, a measure which forms a prominent feature in the programme of the recently established Associated Dairy at Munich, where all the cows are constantly kept under skilled veterinary surveillance, and any that may exhibit the least symptom of tuberculosis are at once weeded out.*

Thus we have abundant proof of the noxious condition of consumptive milk. Comparative pathologists are now agreed on this point; but whether the meat of such diseased animals in the early stages possesses the same infectious properties awaits further investigation. New experiments will be required, and should be instituted at once by State authority, to solve this momentous question pertaining to our health and happiness.

(*To be Continued.*)

---

* *Veterinary Journal*, Feb 1880

